# Assessing the impact of race, social factors and air pollution on birth outcomes: a population-based study

**DOI:** 10.1186/1476-069X-13-4

**Published:** 2014-01-29

**Authors:** Simone C Gray, Sharon E Edwards, Bradley D Schultz, Marie Lynn Miranda

**Affiliations:** 1 U.S. Environmental Protection Agency, Research Triangle Park, North Carolina, USA; 2 Children’s Environmental Health Initiative, School of Natural Resources and Environment, University of Michigan, Ann Arbor, Michigan, USA; 3 Department of Pediatrics, University of Michigan, Ann Arbor, Michigan, USA

**Keywords:** Air pollution, Exposure predictions, Pregnancy outcomes, Socioeconomic status

## Abstract

**Background:**

Both air pollution exposure and socioeconomic status (SES) are important indicators of children’s health. Using highly resolved modeled predictive surfaces, we examine the joint effects of air pollution exposure and measures of SES in a population level analysis of pregnancy outcomes in North Carolina (NC).

**Methods:**

Daily measurements of particulate matter <2.5 μm in aerodynamic diameter (PM_2.5_) and ozone (O_3_) were calculated through a spatial hierarchical Bayesian model which produces census-tract level point predictions. Using multilevel models and NC birth data from 2002–2006, we examine the association between pregnancy averaged PM_2.5_ and O_3_, individual and area-based SES indicators, and birth outcomes.

**Results:**

Maternal race and education, and neighborhood household income were associated with adverse birth outcomes. Predicted concentrations of PM_2.5_ and O_3_ were also associated with an additional effect on reductions in birth weight and increased risks of being born low birth weight and small for gestational age.

**Conclusions:**

This paper builds on and complements previous work on the relationship between pregnancy outcomes and air pollution exposure by using 1) highly resolved air pollution exposure data; 2) a five-year population level sample of pregnancies; and 3) including personal and areal level measures of social determinants of pregnancy outcomes. Results show a stable and negative association between air pollution exposure and adverse birth outcomes. Additionally, the more socially disadvantaged populations are at a greater risk; controlling for both SES and environmental stressors provides a better understanding of the contributing factors to poor children’s health outcomes.

## Background

Both air pollution exposure and socioeconomic status (SES) are important indicators of health, and particularly children’s health. Studies have shown that adverse birth outcomes including low birth weight (LBW; birth weight < 2500 g), small for gestational age (SGA; < 10th percentile of birth weight for gestational age), or preterm birth (PTB; <37 weeks of gestation) have been associated with lower SES [1,2] and maternal exposure to air pollution [3-5]. Adverse birth outcomes are a major public health concern due to the association with increased risk in neonatal mortality and both short- and long-term morbidity [6-9].

Environmental pollutants can have a disparate effect among economically disadvantaged and minority populations [10]. Studies have shown that these groups are also more likely to live in areas with higher levels of pollution, thus potentially detracting from their health [11-14]. A recent population-level analysis in North Carolina, showed that lower SES (income, poverty and education) and higher proportion minority populations (non-Hispanic black [NHB] and Hispanics) were associated with higher levels of particulate matter <2.5 μm in aerodynamic diameter (PM_2.5_) [15]. Studies have also shown that the effects of pregnancy from air pollution exposure on NHB women are greater when compared to the effects on non-Hispanic white (NHW) women [3,11].

Air pollution exposure has also been associated with adverse birth outcomes for both NHB and NHW women [5,16,17]. Results from several studies have shown that higher levels of air pollution exposure have been associated with increased risk in LBW, SGA, and PTB [4,11,18-20]. Studies have also shown that there is a significant and persistent racial and socioeconomic disparity for a number of adverse pregnancy outcomes including infant mortality, LBW, PTB and intrauterine growth restriction (IUGR) [1,21-24]. Historically, infants born to NHB women have consistently lower birth weights than infants born to NHW women [25]. In 2010, the PTB rate for NHB was 16.9% compared to 10.8% for NHW and 11.8% for Hispanics [26]. For LBW, the prevalence for NHB was 13.5% compared to 7.1% and 7.0% for NHW and Hispanics [26]. Mothers living in low income neighborhoods and with lower levels of education are also at an increased risk for adverse birth outcomes [1,2,26,27].

Evidence has shown that there exists a relationship between SES and pregnancy outcomes [11,23,28,29], as well as SES and environmental exposures [30,31]. Because of these interrelated factors, care should be taken in trying to understand how both SES and air pollution jointly affect adverse birth outcomes. While individual-level social characteristics have been identified as risk factors for pregnancy outcomes, studies also suggest that neighborhood-level SES measures are also important [2,19]. Jointly studying the effects of individual- and area-level SES measures with air pollution on birth weight provides a more complete story of this important relationship.

As exact measures of personal air pollution exposure are both difficult and expensive to obtain, health studies often use measurements obtained from monitoring stations as surrogates for individual exposure assessment [3,5]. These collected measurements are obtained from a sparse network of monitoring stations and are not always collected on a daily basis, leading to large amounts of missing data. Alternatively, proximity to major roadways has also been used as a metric for individual air pollution exposures due to the large contribution of traffic emissions to ambient air pollution [4,19,32,33]. Other studies have explored the use of modeling techniques for estimating personal exposure including the Community Multi-Scale Air Quality Model (CMAQ), the probabilistic NAAQS Exposure Model (pCNEM), and the Stochastic Human Exposure and Dose Simulation Model (SHEDS-PM) [34-36]. In this study, we address the concern of missing data, both spatially and temporally, by using daily modeled predicted surfaces of air pollution as exposure inputs.

Our objective was to conduct a population level study of all pregnancies in North Carolina (NC) over a five-year period focusing on the effects of SES and air pollution. We assess the relationship between birth weight (BWT), LBW, SGA, and PTB and modeled air pollution measures. We use modeled predictive surfaces of O_3_ and PM_2.5_ to get daily concentration estimates at the census-tract level. This highly spatially resolved concentration metric is available for the entire state of NC, which allows us to include women that would typically be excluded from analyses that use only monitoring networks as exposure surrogates. We obtain personal data from the North Carolina Detailed Birth Records (NCDBR) and neighborhood SES data from the US Census. We use both individual- and area-level measures of SES to capture the effects of the individual SES measures, as well as any additional residual area-level effects that may exist.

## Methods

### Study population

NCDBR data were obtained from the North Carolina State Center for Health Statistics for the years 2002–2006. The NCDBR contains birth outcomes, as well as maternal characteristics and demographics, for all registered live births in NC (n=604,757). Infant characteristics obtained from the birth records included clinical estimate of gestational age (weeks), infant sex, BWT, indication of congenital anomalies, plurality, and date of birth. The maternal characteristics recorded in the NCDBR included residential address at time of birth, age, marital status, education, race and ethnicity, tobacco use, parity, and the trimester in which prenatal care began.

To link births from the NCDBR to the air pollution data, we used ArcGIS 9.3 software to geocode individual maternal residential street addresses at the street block level (ESRI, Redlands, CA). Approximately 14% of the total births could not be successfully geocoded due to poor address information and were omitted from further analyses. We excluded multiple births (3.4%), and infants with diagnosed congenital anomalies at the time of birth (0.8%). These exclusions were chosen as we sought to focus on those pregnancies that could reasonably be expected to go to term and deliver at a normal BWT. We also excluded women < 15 and > 44 years of age (0.3%) with more than 4 previous deliveries (3.8%). Since 96% of the women in the dataset self-declared as NHW, NHB, or Hispanic, we excluded women of other races/ethnicities due to the small numbers in other minority groups. We excluded births with gestation < 24 and > 42 weeks (0.1%), BWT < 400g (0.2%), and mothers with any missing data on covariates (1.0%), leaving 457,642 births. This study was conducted in accordance with a human subjects research protocol approved by the Duke University and University of Michigan Institutional Review Boards.

### Exposure measure

Air pollution data from 2001–2006 were used as surrogate exposure measurements for this study, as exposure during pregnancy for some births in 2002 began in 2001. Using a Bayesian space-time downscaling fusion model, daily predicted PM_2.5_ (daily average in μg/m^3^) and O_3_ (daily 8-hr maximum in ppb) surfaces were obtained at the census tract level for the entire State of NC. Archived daily downscaler surfaces for 2001–2008 are available at: http://www.epa.gov/esd/land-sci/lcb/lcb_faqsd.html. The downscaling fusion model uses input data from two sources: (1) monitoring networks and (2) numerical models. The air quality monitoring data came from the US Environmental Protection Agency Air Quality System repository database, and the numerical output came from the Models-3/Community Multiscale Air Quality (CMAQ; http://www.epa.gov/AMD/Research/RIA/cmaq.html). Further details and descriptions of the modeling approach can be found in Berrocal et al. [37].

To estimate air pollution exposure, we linked births to the census tract corresponding to each mother’s residence at the time of delivery. The time period of exposure for each birth was calculated using the date of birth and the weeks of gestation at delivery, as recorded in the NCDBR. Using daily estimated pollution levels from the fused data, we calculated average exposure for the entire pregnancy. The high spatial and temporal resolution of the fused data provides exposure estimates for all of the successfully geocoded birth records, with no missing values for any days during the exposure window.

### SES data

We use individual measures of SES from the NCDBR and an areal measure of household median income from the 2000 US Census. The size of census tracts in North Carolina ranges from 0.4 to 3529.6 sq km with a mean of 87.8 and a standard deviation (SD) of 171.3. Population density ranged from 2 to 4380 people per sq km with a mean of 442 and SD of 550. From the NCDBR, maternal education was categorized by years of completion as < 9 (middle school), 9–11 (some high school), 12 (completed high school), 13–15 (some college), and > 15 years (completed college). We categorized the census tract-level household median income into tertiles as low, moderate, and high.

### Statistical analysis

Linear and logistic mixed regression modeling were used to determine the association between exposure to the pollutants of interest, O_3_ and PM_2.5_, and an array of pregnancy outcomes. Using BWT (g) as a continuous outcome variable, we examined the effect of average O_3_ and PM_2.5_ over the entire pregnancy while controlling for gestational age (24–34, 35–36, 37–42 weeks), maternal race/ethnicity (NHB, NHW, or Hispanic), maternal education, maternal age at delivery (15–19, 20–24, 25–29, 30–34, 35–39, 40–44 years), trimester prenatal care began, tobacco use during pregnancy (yes or no), marital status at delivery (married or unmarried), year of birth, parity (first birth, yes or no), infant sex, and census tract-level median household income. We also examined the effects of air pollution exposure on the binary outcomes of LBW, SGA, and PTB. SGA was calculated by comparing each record’s birth weight and gestation age to a sex-specific, national reference distribution of birth weights for a given gestational age at delivery. Census tracts were specified as a random effect to account for neighborhood level correlation. Analyses were performed using SAS 9.3 (SAS Institute, Cary, NC).

## Results

### Descriptive analysis

Our analysis included 457,642 births after the exclusion criteria were applied. In the study population, 7.3% of births were LBW, 10.2% were SGA, and 9.8% were PTB. Table 1 shows the summary statistics for the entire study population and those with adverse birth outcomes. Our overall study population consisted mainly of women who were NHW (62%), married (64%), under 30 years of age (66%), with at least a high school education (79%), and living in moderate to high income census tracts (75%). Approximately 12% of the women reported smoking during pregnancy.

**Table 1:** Demographics and clinical metrics of the study population overall and among births with adverse birth outcomes

	**All births (n= 457 642)**	**LBW (n = 30 458)**	**SGA (n = 42 663)**	**PTB (n = 40 746)**
Mean BWT in g (SD)	3320.8 (573.7)	2003.1 (508.0)	2534.1 (387.2)	2392.6 (717.0)
Gestational age (%)				
24-34 weeks	3.3	43.3	4.3	37.2
35-36 weeks	5.6	25.0	7.6	62.8
37-42 weeks	91.1	31.7	88.0	N/A
Infant sex male (%)	51.2	46.9	50.3	53.2
First born (%)	42.7	50.9	52.5	45.7
Trimester prenatal care began (%)				
First	84.9	81.0	79.6	83.2
Second	12.6	15.0	16.7	12.4
Third	1.9	1.9	2.6	2.1
None	0.6	2.1	1.1	2.3
Year of birth (%)				
2002	19.2	19.1	19.0	19.0
2003	19.3	19.4	19.3	19.5
2004	19.9	19.5	19.2	20.0
2005	20.4	20.5	20.6	20.5
2006	21.1	21.5	22.0	20.9
Race/ethnicity (%)				
Non-hispanic white	62.4	39.0	48.2	57.5
Non-hispanic black	22.8	50.2	37.7	31.2
Hispanic	14.8	10.8	14.1	11.3
Maternal education (%)				
<9 years	6.2	5.6	6.8	5.3
9-11 years	15.0	21.2	22.5	17.4
12 years	28.4	33.1	33.3	30.7
13-15 years	22.4	21.6	20.0	23.5
>15 years	28.1	18.5	17.4	23.1
Maternal age (%)				
15-19 years	11.4	16.4	17.8	13.5
20-24 years	27.2	30.4	33.0	27.6
25-29 years	27.2	23.8	23.7	25.3
30-34 years	22.7	18.3	16.4	21.0
35-39 years	9.9	9.1	7.6	10.5
40-44 years	1.7	2.0	1.6	2.2
Tobacco use (%)	11.7	21.0	22.2	15.5
Married (%)	64.4	49.7	48.1	58.1
Household median income ($)				
Mean (SD)	424000 (15490)	39187 (13753)	39284 (14083)	40605 (14403)
Household median income (%)				
Low	24.4	30.9	31.2	27.6
Moderate	32.5	34.2	33.9	33.8
High	43.1	34.9	34.9	38.6
PM_2.5_ (μg/m^3^)				
Mean (SD)	13.6 (1.7)	13.6 (1.8)	13.7 (1.7)	13.6 (1.8)
Interquartile range	2.0	2.3	2.0	2.3
O_3_ in ppb				
Mean (SD)	43.2 (4.0)	43.3 (5.0)	43.3 (4.1)	43.2 (5.0)
Interquartile range	5.9	7.4	6.0	7.5

Among births incurring a poor birth outcome, the proportion of births to mothers who were NHB and living in low household median income census tracts were greater than in the overall population of women. The largest difference between the demographic composition of the overall population and among births with adverse outcomes was seen among LBW births, where the proportion of NHB mothers rises from 23% in the general population to 50% among LBW births. Higher rates of firstborn births, women receiving no prenatal care, those who smoked during pregnancy, and mothers with lower educational attainment, all characteristics associated with poor birth outcomes, were seen among the poor birth outcomes subgroups compared to all births (Table 1).

Women with adverse birth outcomes had similar mean PM_2.5_ and O_3_ concentrations during pregnancy as the overall study population (Table 1). Mean air pollution levels for PM_2.5_ and O_3_ remain relatively consistent across race/ethnicity and the SES variables (results not shown). We observe a pairwise statistically significant relationship among the race/ethnicity, maternal education, and household median income variables, with p<.0001 (data not shown).

### PM_2.5_ Multivariable analyses

We calculated odds ratios (ORs) and 95% confidence intervals (CI) for all birth outcomes. Table 2 displays the associations between race, SES, and PM_2.5_ exposure and birth outcomes. NHB and Hispanic mothers were more likely to have infants born with lower birth weight when compared with NHW mothers (−187.5g, 95% CI: -183.6 to −191.4 and −46.8g, 95% CI: -41.8 to −51.7, respectively). Infants born to NHB mothers were also at an increased odds of being born LBW (adjusted OR 2.13, 95% CI: 2.05 to 2.22), PTB (adjusted OR 1.46, 95% CI: 1.42 to 1.50), and SGA (adjusted OR 2.18, 95% CI: 2.12 to 2.24). In models for all outcomes, highly educated mothers and those living in high SES neighborhoods had better birth outcomes compared to mothers with only a high school level education and those living in moderate income neighborhoods (Table 2).

**Table 2:** **Effects of Race/Ethnicity, SES and Fused PM**_**2.5 **_**on adverse birth outcomes**^**a**^

	**Birth weight**	**LBW**	**SGA**	**PTB**
	**Change in grams (95% CI)**	**OR (95% CI)**	**OR (95% CI)**	**OR (95% CI)**
% Race/ethnicity				
NHW	Reference	Reference	Reference	Reference
NHB	−187.5 (−191.4, −183.6)	2.13 (2.05, 2.22)	2.18 (2.12, 2.24)	1.46 (1.42, 1.50)
Hispanic	−46.8 (−51.7, −41.9)	0.99 (0.93, 1.05)	1.21 (1.17, 1.26)	0.78 (0.75, 0.81)
% Maternal education (years)				
<9	−28.3 (−35.1, −21.5)	1.17 (1.08, 1.26)	1.14 (1.09, 1.20)	1.04 (0.99, 1.10)
9-11	−33.1 (−37.7, −28.5)	1.19 (1.14, 1.25)	1.15 (1.12, 1.19)	1.08 (1.05, 1.12)
12	Reference	Reference	Reference	Reference
13-15	18.5 (14.5, 22.5)	0.82 (0.79, 0.86)	0.85 (0.82, 0.87)	0.98 (0.95, 1.01)
>15	31.6 (27.2, 36.0)	0.72 (0.68, 0.75)	0.72 (0.69, 0.75)	0.78 (0.75, 0.80)
% Household median income				
Low	−7.5 (−12.5, −2.5)	1.04 (1.00, 1.08)	1.04 (1.00, 1.07)	1.01 (0.98, 1.04)
Moderate	Reference	Reference	Reference	Reference
High	11.4 (6.4, 15.8)	0.92 (0.88, 0.95)	0.93 (0.90, 0.96)	0.90 (0.87, 0.93)
PM_2.5_ per IQR increase	−3.13 (−3.34, −2.93)	1.02 (0.99, 1.04)	1.03 (1.02, 1.05)	1.01 (0.99, 1.02)

^a^Additional covaraites include gestational age (weeks), maternal age, trimester prenatal care began, tobacco use during pregnancy, marital status, year of birth, parity, and infant sex.

For the continuous BWT models, PM_2.5_ was associated with a small but significant reduction in BWT. An inter-quartile range (IQR) increase in PM_2.5_ during the entire gestational period reduced BWT by 3.1 g (95% CI: 3.0 to 3.2). An IQR increase in maternal exposure to PM_2.5_ was significantly associated with increased odds of being SGA (adjusted OR 1.03, 95% CI: 1.02 to 1.05), but not LBW or PTB.

### O_3_ Multivariable analyses

Table 3 displays the associations between race, SES, and O_3_ exposure and birth outcomes. Race and SES followed similar patterns to those seen in the PM_2.5_ models. NHB and Hispanic mothers were more likely to have infants born with lower birth weight when compared with NHW mothers (−188.2g, 95% CI: -184.3 to −192.1 and −47.3g, 95% CI: -42.4 to −52.3, respectively). Infants born to NHB mothers were also at increased odds of being born LBW (adjusted OR 2.15, 95% CI: 2.06 to 2.23), PTB (adjusted OR 1.46, 95% CI: 1.43 to 1.51), and SGA (adjusted OR 2.20, 95% CI: 2.14 to 2.26). Across all models, highly educated mothers and those living in census tracts with high household median income had better birth outcomes when compared to mothers with only a high school level education and those living in moderate income neighborhoods (Table 3).

**Table 3:** **Effects of race/ethnicity, SES and fused O**_**3 **_**on adverse birth outcomes**^**a**^

	**Birth weight**	**LBW**	**SGA**	**PTB**
	**Change in grams (95% CI)**	**OR (95% CI)**	**OR (95% CI)**	**OR (95% CI)**
% Race/ethnicity				
NHW	Reference	Reference	Reference	Reference
NHB	−188.2 (−192.1, −184.3)	2.15 (2.06, 2.23)	2.20 (2.14, 2.26)	1.46 (1.43, 1.51)
Hispanic	−47.3 (−52.3, −42.4)	0.99 (0.93, 1.05)	1.22 (1.18, 1.27)	0.78 (0.75, 0.81)
% Maternal education (years)				
<9	−28.6 (−35.4, −21.8)	1.17 (1.08, 1.26)	1.15 (1.09, 1.20)	1.04 (0.99, 1.10)
9-11	−33.1 (−37.7, −28.5)	1.19 (1.14, 1.25)	1.15 (1.12, 1.19)	1.08 (1.05, 1.12)
12	Reference	Reference	Reference	Reference
13-15	18.5 (14.5, 22.5)	0.82 (0.79, 0.86)	0.85 (0.82, 0.87)	0.98 (0.95, 1.01)
>15	31.1 (26.7, 35.5)	0.72 (0.68, 0.76)	0.72 (0.70, 0.75)	0.78 (0.75, 0.80)
% Household median income				
Low	−7.6 (−12.6, −2.6)	1.04 (1.00, 1.09)	1.03 (1.00, 1.07)	1.01 (0.98, 1.04)
Moderate	Reference	Reference	Reference	Reference
High	10.0 (5.4, 14.6)	0.92 (0.88, 0.96)	0.94 (0.81, 0.97)	0.90 (0.87, 0.93)
O_3_ per IQR increase	−7.4 (−9.5, −5.2)	1.06 (1.03, 1.09)	1.04 (1.03, 1.06)	1.02 (0.99, 1.04)

^a^Additional covariates include gestational age (weeks), maternal age, trimester prenatal care began, tobacco use during pregnancy, marital status, year of birth, parity, and infant sex.

Comparable to the PM_2.5_ results, the models for the continuous BWT outcomes showed a significant negative association with O_3_. An IQR increase in O_3_ during the entire gestational period reduced BWT by 7.4 g (95% CI: 5.2 to 9.5). Additionally, an IQR increase in maternal exposure to O_3_ was significantly associated with increased odds of being SGA (adjusted OR 1.04, 95% CI: 1.03 to 1.06) and LBW (adjusted OR 1.06, 95% CI: 1.03 to 1.09), but not PTB.

### Sensitivity analyses

Sensitivity analyses controlling for seasonality did not reveal season to be an important predictor of outcomes. Models adjusting for urban and rural indicators also did not modify the reported results. Interaction terms of SES and air pollution did not produce significant results for any of the adverse birth outcomes.

## Discussion

We examined the effects of maternal exposure to PM_2.5_ and O_3_ and measures of SES on birth outcomes in NC. Our results indicate that both O_3_ and PM_2.5_ are associated with adverse birth outcomes, specifically LBW and SGA. As expected, individual and neighborhood SES also contribute to adverse birth outcomes. We found that NHB mothers and women with lower educational attainment were generally at an increased risk of having a poor birth outcome when compared to NHW mother and those with higher levels of education. Mothers living in census tracts with lower household median income were also at an increased risk of adverse pregnancy outcomes. Air pollution exposure contributed an additional harmful effect on pregnancy after controlling for race and individual and area-level SES.

Our findings on the association between adverse birth outcomes and PM_2.5_ are consistent with previous studies [3,5,20]. Using monitoring data from Connecticut and Massachusetts, Bell et al. [3] showed reductions in BWT per IQR increase in PM_2.5_ during the entire pregnancy. In contrast to our study results, Bell et al. also showed increased odds of being born LBW for PM_2.5_. Gray et al. [5] used monitoring data in a previous North Carolina study and showed reductions in BWT for PM_2.5_.

While a number of studies have examined the relationship between particulate matter and pregnancy, fewer studies have focused on O_3_, and fewer still have found a consistent and significant relationship with adverse pregnancy outcomes [20,38,39]. A recent study in Texas found a negative effect on BWT per IQR increase of O_3_ during the entire gestational period [40]. Salam et al. [35] showed similar results for O_3_ and BWT in a California study. Liu et al. [41] found no statistically significant results for O_3_ and PTB or LBW in Vancouver, Canada.

This study is not without limitations. We acknowledge that the modeled concentration data for O_3_ and PM_2.5_ are not explicit measures of maternal exposure to pollution, as they do not take into account daily activity patterns, indoor sources, or ventilation conditions. We also used maternal address at the time of delivery, which may not necessarily be the address during the entire pregnancy. Data on residential mobility were not available for this study. These results may also be affected by residual confounding as there are several other factors we were unable to control for that can contribute to pregnancy outcomes, including access to healthcare, pre-pregnancy weight, maternal height, and nutrition.

One of the key strengths of this study is the availability of exposure data for the entire State of NC with no missing days of exposure data. In environmental health studies, air pollution measurements from a sparse network of monitoring stations or proximity to major roadways are commonly used as a proxy for personal exposure. The fine spatial and temporal resolution of the modeled air pollution data allowed us to obtain estimates for times and locations where exposure data are not otherwise available. With air pollution estimates for the entire state, the analyses were performed at the population level rather than in a non-randomly restricted subset of the population based on proximity to environmental hazards or monitoring stations.

Previous studies have used hierarchical models to examine the relationship between individual and local SES, pollution, and adverse birth outcomes, but many focus on traffic-related pollution, proximity to highways, lead, or sulfur dioxide [18,19,42,43]. Our study, on the other hand, uses a multi-level structure to incorporate individual and neighborhood level SES factors along with modeled concentration data for PM_2.5_ and O_3_.

The biological mechanisms through which air pollution may affect pregnancy are still unclear. Air pollution may affect birth outcomes through multiple pathways including oxidative stress, blood flow, placental formation and function, and inflammatory responses [44]. Additional factors that can also influence this relationship include the timing of the air pollution exposure, the pollutant and the birth outcome of interest [45]. Recent studies have examined the relationship between air pollution and pregnancy outcomes using animal models. Evidence suggests that prenatal exposures to air pollution in mice can impair fetal lung growth and increase both airway hyper-responsiveness and infiltration of inflammatory cells [46,47]. Another mouse study showed that exposure during pregnancy to O_3_ affected both lung function and reproductive outcomes in the offspring [48]. Increased O_3_ exposure was associated with decreases in the number of viable pregnancies and the body weight in the surviving offspring [48]. More work on examing the biological pathways is critical in understanding the association between air pollution and adverse birth outcomes.

## Conclusions

This study on infant health uses modeled state-wide measurements of air pollution along with individual and neighborhood SES in a hierarchical model on pregnancy outcomes. Our results show that despite NC’s consistent attainment of federal air quality standards, there is a stable and negative association between air pollution exposure and adverse birth outcomes. Additionally, the more socially disadvantaged populations are at a greater risk, and controlling for both SES and environmental stressors provides a better understanding of the factors contributing to reproductive health.

## Abbreviations

BWT: Birth weight; CI: Confidence interval; CMAQ: Community Multi-Scale Air Quality Model; IUGR: Intrauterine growth restriction; LBW: Low birth weigh; NC: North Carolina; NCDBR: North Carolina Detailed Birth Records; NHB: Non-Hispanic Black; NHW: Non-Hispanic White; O3: Ozone; OR: Odds ratio; pCNEM: Probabilistic NAAQS Exposure Model; PM2.5: Particulate matter <2.5 μm in aerodynamic diameter; PTB: Preterm birth; SES: Socioeconomic status; SGA: Small for gestational age; SHEDS-PM: Stochastic human exposure and dose simulation model.

## Competing interests

The authors declare that they have no competing interests.

## Authors’ contributions

SCG participated in the original study design, carried out the primary data analysis, and prepared the first draft of the manuscript. SEE coordinated the data collection and participated in the manuscript preparation. BDS and MLM made substantial contributions to the design and direction of the study. All authors assisted with drafting and revising the manuscript. All authors have read and approved the final manuscript.
